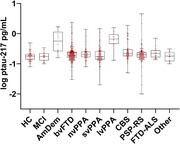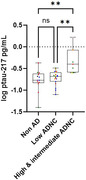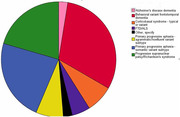# Clinical performance of plasma P‐tau217 for the identification of primary Alzheimer’s disease pathology or co‐pathology in sporadic frontotemporal dementia

**DOI:** 10.1002/alz.089822

**Published:** 2025-01-09

**Authors:** Igor Prufer Q C Araujo, Julio C. Rojas, Lawren VandeVrede, Peter A. Ljubenkov, Hilary W. Heuer, Bruce L. Miller, William W. Seeley, Adam M. Staffaroni, Binita Rajbanshi, Argentina Lario Lago, Elisabeth H Thijssen, Gianina Toller, Nicholas Proctor, Leah K. Forsberg, Leah K. Forsberg, Danielle Brushaber, Eliana Marisa Ramos, Giovanni Coppola, Brian Appleby, Yvette M. Bordelon, Hugo Botha, Brad C Dickerson, Dennis W. Dickson, Kimiko Domoto‐Reilly, Anne M. Fagan, Julie A. Fields, Jamie C Fong, Tatiana M. Foroud, Douglas R. Galasko, Ralitza H. Gavrilova, Daniel H. Geschwind, Jill S Goldman, Nupur Ghoshal, Neill R Graff‐Radford, Jonathan Graff‐Radford, Ian Grant, Murray Grossman, Ging‐Yuek Robin Hsiung, Eric J. Huang, Edward D. Huey, David J Irwin, David T. Jones, David S. Knopman, John Kornak, Kejal Kantarci, Walter K. Kremers, Maria I. Lapid, Gabriel C Leger, Irene Litvan, Diane E. Lucente, Ian R MacKenzie, Joseph C. Masdeu, Corey T McMillan, Mario F. Mendez, Toji Miyagawa, Chiadi U. Onyike, Belen Pascual, Otto Pedraza, Leonard Petrucelli, Rosa Rademakers, Katherine P. Rankin, Katya Rascovsky, Jessica E Rexach, Aaron Ritter, Erik D Roberson, Maria Carmela Tartaglia, Arthur W. Toga, Sandra Weintraub, Bonnie Wong, Zbigniew Wszolek, Jeffrey L. Dage, Brad F. Boeve, Howard J. Rosen, Adam L. Boxer

**Affiliations:** ^1^ Memory and Aging Center, Weill Institute for Neurosciences, University of California San Francisco, San Francisco, CA USA; ^2^ Memory and Aging Center, Weill Institute for Neurosciences, University of California, San Francisco, San Francisco, CA USA; ^3^ UCSF Alzheimer's Disease Research Center, San Francisco, CA USA; ^4^ University of California San Francisco, San Francisco, CA USA; ^5^ University of California San Francisco (UCSF), San Francisco, CA USA; ^6^ Weill Institute for Neurosciences and Memory and Aging Center, Department of Neurology, University of California, San Francisco, CA USA; ^7^ Memory and Aging Center, UCSF Weill Institute for Neurosciences, University of California San Francisco, San Francisco, CA USA; ^8^ Memory and Aging Center, UCSF Weill Institute for Neurosciences, University of California, San Francisco, San Francisco, CA USA; ^9^ Memory and Aging Center, Weill Institute for Neurosciences, University of California San Francisco, San Francisco, CA, USA, San Francisco, CA USA; ^10^ University of California, San Francisco, San Francisco, CA USA; ^11^ Neurochemistry Laboratory, Department of Clinical Chemistry, Vrije Universiteit Amsterdam, Amsterdam UMC, Amsterdam Netherlands; ^12^ Memory and Aging Center, University of California San Francisco, San Francisco, CA USA; ^13^ Kantonsspital, St. Gallen, St. Gallen Switzerland; ^14^ Eli Lilly and Company, Indianapolis, IN USA; ^15^ Department of Neurology, Mayo Clinic, Rochester, MN USA; ^16^ Mayo Clinic, Rochester, MN USA; ^17^ University of California, Los Angeles, Los Angeles, CA USA; ^18^ Case Western Reserve University, Cleveland, OH USA; ^19^ University of California Los Angeles, Los Angeles, CA USA; ^20^ Massachusetts General Hospital, Charlestown, MA USA; ^21^ Department of Neuroscience, Mayo Clinic, Jacksonville, FL USA; ^22^ University of Washington, Seattle, WA USA; ^23^ Department of Neurology, Washington University School of Medicine, St. Louis, MO USA; ^24^ Department of Psychiatry and Psychology, Mayo Clinic, Rochester, MN USA; ^25^ National Centralized Repository for Alzheimer's Disease and Related Dementias (NCRAD), Indianapolis, IN USA; ^26^ University of California, San Diego, La Jolla, CA USA; ^27^ University of California, Los Angeles School of Medicine, Los Angeles, CA USA; ^28^ Columbia University, New York, NY USA; ^29^ Washington University, Saint Louis, MO USA; ^30^ Department of Neurology, Mayo Clinic, Jacksonville, FL USA; ^31^ Northwestern University, Chicago, IL USA; ^32^ University of Pennsylvania, Philadelphia, PA USA; ^33^ University of British Columbia, Vancouver, BC Canada; ^34^ Perelman School of Medicine, University of Pennsylvania, Philadelphia, PA USA; ^35^ University of California San Diego, La Jolla, CA USA; ^36^ Massachusetts General Hospital, Boston, MA USA; ^37^ The University of British Columbia, Vancouver, BC Canada; ^38^ Houston Methodist Research Institute, Houston, TX USA; ^39^ The University of Tokyo, Tokyo Japan; ^40^ Johns Hopkins University School of Medicine, Baltimore, MD USA; ^41^ Houston Methodist Neurological Institute, Houston, TX USA; ^42^ Mayo Clinic, Jacksonville, FL USA; ^43^ VIB‐UAntwerp Center for Molecular Neurology, University of Antwerp, Antwerp, Antwerp Belgium; ^44^ Program in Neurogenetics, Department of Neurology, David Geffen School of Medicine, University of California Los Angeles, Los Angeles, CA USA; ^45^ Cleveland Clinic Lou Ruvo Center for Brain Health, Las Vegas, NV USA; ^46^ University of Alabama at Birmingham, Birmingham, AL USA; ^47^ University of Toronto, Toronto, ON Canada; ^48^ University of Southern California, Los Angeles, CA USA; ^49^ Northwestern University Feinberg School of Medicine, Chicago, IL USA; ^50^ Massachusetts General Hospital, Harvard Medical School, Boston, MA USA

## Abstract

**Background:**

As new anti‐amyloid immunotherapies emerge for Alzheimer’s disease (AD), it is clear that early diagnosis of AD pathology is crucial for treatment success. This can be challenging in atypical presentations of AD and, together with our reliance on CSF or PET scans, can, at times, lead to delayed diagnosis. Here, we further explore the possible role of plasma tau phosphorylated at threonine 217 (P‐tau217) for the detection of primary AD or AD co‐pathology when frontotemporal dementia spectrum disorders are the main clinical presentation.

**Method:**

Participants were recruited through ALLFTD, a North American multisite research consortium for the longitudinal assessment of frontotemporal lobar degeneration. After excluding cases with known FTD‐causing mutations, 573 participants with sporadic disease, mild cognitive/behavioral impairment, or healthy familial controls evaluated between 2014 and 2023 were included in this analysis (45.8% female, median age 66 years). 39 of those cases underwent neuropathological evaluation. Plasma P‐tau217 was measured with the Eli Lilly electrochemiluminescence‐based assay. P‐tau217 concentrations were compared by phenotype, disease severity, genotype, and, when available, neuropathological diagnosis with non‐parametric tests. Its diagnostic performance was tested with ROC curves. Associations between plasma P‐tau217 concentrations and clinical scales of global cognition, memory, executive function, motor function, and social cognition with linear regressions and non‐parametric tests.

**Result:**

Plasma P‐tau217 concentrations were higher in logopenic variant primary progressive aphasia (lvPPA) and amnestic dementia (AmDem), compared to participants with normal cognition or other FTD phenotypes. P‐tau217 concentrations were higher in APOE e4 carriers, compared to non‐carriers, regardless of phenotype, but were not affected by disease severity. P‐tau217 concentrations showed associations with clinical measures of global cognition, memory, and executive function, but had no associations with measures of motor function or social cognition. Plasma P‐tau217 concentrations were elevated in high and intermediate ADNC scoring, or more advanced Braak stages, even when AD was not the primary pathology.

**Conclusion:**

Even when FTD is suspected, high plasma p‐tau 217 concentrations are strongly associated with underlying AD as primary pathology or contributing co‐pathology. Plasma P‐tau217 could be a tool to support the development of disease‐modifying therapies for atypical Alzheimer’s disease presentations or for FTD cases with Alzheimer’s disease co‐pathology.